# Anesthesia-related Safety Profile of a High-energy Ultrasonic Scalpel in Oropharyngeal and Laryngeal Surgery: An Ex Vivo Model

**DOI:** 10.7759/cureus.5266

**Published:** 2019-07-29

**Authors:** Nikolay R Sapundzhiev, Georgi Davidov, Viliyan Platikanov, George S Stoyanov, Valentin Ignatov

**Affiliations:** 1 Otolaryngology, Medical University of Varna, Varna, BGR; 2 Otolaryngology, St. Marina University Hospital, Varna, BGR; 3 Anesthesiology, St. Marina University Hospital, Varna, BGR; 4 Pathology, Medical University of Varna, Varna, BGR; 5 Surgery, St. Marina University Hospital, Varna, BGR

**Keywords:** ultrasonic scalpel, laryngeal surgery, safety, ex vivo model

## Abstract

Introduction

The aim of this study was to evaluate the fire risks associated with a harmonic scalpel, with an established avian model simulating oropharyngeal/laryngeal surgery.

Methods

A standard polyvinyl-chloride (PVC) endotracheal tube (ETT) was inserted into a degutted, whole raw chicken through which 100% oxygen was piped at 10 L/min. The inflated cuff of the tube was grasped and sectioned with the jaws of a standard high-power ultrasonic dissection system Ultracision (Ethicon Endosurgery, Cincinnati, Ohio, USA). Then, the whole ETT was grasped and cut, leaving the device in contact with the ETT for two more minutes. In a second step under the same conditions, an electrosurgical device was placed into the chicken cavity and activated at the chicken tissue near the ETT at a setting of 20 W. All trials were repeated to ensure accuracy.

Results

No ignition could be produced with the harmonic scalpel under any operation mode settings. In all cases, the ETT was cut through with some fumes and brown discoloration at the site of contact. The electrosurgical device easily caused flash ignition within seconds.

Conclusion

The harmonic scalpel appears to be a safer tool than electrosurgical devices in the setting of open cavity surgery in oxygen-enriched environments with respect to the presence of flammable medical PVC devices as ETT or catheters.

## Introduction

High-energy surgical tools and light sources are a well-recognized factor for fires in the operating field. The head and neck region seem to be at high risk for such incidents, accounting for more than 70% of external fires and more than 30% of internal ones [1]. Surgical equipment used close to endotracheal tubes (ETTs) may be associated with particular risks and both the surgeon and the anesthesiologist should be aware of them [2]. With the abundance of high-energy surgical tools, flammable surgical materials, and the oxygen-enriched surgical field beneath the drapes and in the airway, the hazard of a surgical fire is clearly still with us, especially in surgery of the pharynx and larynx [3]. In the last decades, harmonic scalpels have been in use for various interventions in otorhinolaryngology, particularly in the pharynx and surrounding structures [4-9], which may eventually endanger the ETT. Only one report so far briefly discusses its safety with regard to the ignition of the ETT in the presence of oxygen during tracheotomies and cricothyroidotomies [10].

With this background, the aim of the present study was to evaluate the fire risk for a commercially available harmonic scalpel (Ultracision Harmonic Scalpel Generator 300 with Harmonic Ace forceps; Ethicon Endosurgery, Cincinnati, Ohio, USA) in an established ex vivo model of oropharyngeal and laryngeal surgery [11].

## Materials and methods

The tests were carried out in a standard operating room (OR). Extra care was taken to ensure there were no other gases (especially flammable) in the atmosphere apart of standard air, by ventilating well for a period of more than 30 minutes. The high-energy ultrasonic dissection system tested was UltraCision (Ethicon Endosurgery, Cincinnati, Ohio, USA), which consists of a high-frequency vibration generator (300 series) and a handpiece with cable (Harmonic Ace Shears, Ethicon). In this experiment, we used generator power level five. The comparator was an electrocautery Bovie unit (Valleylab Force 40, Valleylab, Inc., CO, US), which was operated in monopolar mode with a power setting of 20 Watts. A test was classified as ‘‘negative’’ if no ignition could be obtained before creating a substantial opening on the ETT, comprising at least half of its circumference or its complete cutting and for the next two minutes while holding the activated (but not clamped) Harmonic Ace device in contact with the ETT. Remote measurement of the local temperatures was attempted with an infrared thermometer (FLUKE 65, Fluke UK Ltd, Norfolk, United Kingdom).

Phase one: direct test

Polyvinyl chloride (PVC) ETTs 3.0 (Beromed GmbH Hospital Products, Berlin, Germany) were connected to the available standard anesthesia delivery device and flown with 100% oxygen at 10 L/min. Extra care was taken to ensure the possibility for emergency oxygen cut-off by clamping/separating the tubes about two meters away from the PVC-ETT. Initially, the inflated cuff of the tube was grasped and sectioned with the harmonic (Ethicon G300 Ultracision Harmonic Scalpel). This was achieved within two seconds, immediately after the activation of the powered instrument. No signs of ignition were observed. Then, the tip of the tube was grasped with the powered instrument 2 mm - 3mm back from the distal opening. The device was activated and the ETT was cut through. The device was held into contact with the tube for two more minutes under the same oxygen flow. The procedure was repeated on the same ETT three to four times each time some 4 mm - 5 mm proximally.

Phase two: physical model

The Roy and Smith model for oropharyngeal fires was adopted, which uses a degutted, whole raw chicken with an incomplete occlusion of the cranial end, allowing for the insertion of a standard 6.0 PVC-ETT 11. Tonsil sponges were omitted, as they are not currently used/available in our institution/country and are judged to be only uncommon fuel [12]. For each trial, 100% oxygen was piped through the PVC-ETT at 10 L/min through a standard anesthesia delivery device. The cavity of the chicken was pre-oxygenated for several seconds by flowing oxygen through the ETT. Next, a tissue section was performed with the Harmonic Ace Shears near the tip of the ETT. Then, the ETT was directly attacked with the surgical tool, as described above: first the cuff and then the body of the tube at several locations, starting at its tip and going proximally.

Phase three: verification of the physical model

After negative testing with the Ultracision Harmonic forceps, the chicken was grounded to the electrosurgical device ground pad. The ETT tube was replaced with a new one with an inflated cuff. Again, 100% oxygen was piped through the PVC-ETT at 10 L/min through a standard anesthesia delivery device. The electrosurgical device was activated in the preoxygenated cavity of the chicken at tissues close to the tip of the ETT.

## Results

Phase one: direct test

No ignition or sustained fire could be produced at any location of the ETT. Only a touch of the activated tool was enough to damage the cuff of the ETT. While cutting the body of the ETT with the Harmonic Ace, some visible aerosols developed. We are unable to state if this was smoke from non-flaming burning (smoldering or glowing) or any other form of colloid of solid or liquid PVC particles. Some brown discoloration appeared at the contact point between the harmonic and the ETT. An attempt at remote temperature measurement failed. Holding the closed shears in contact with the ETT after having cut it for the control for two minutes led to the damage of the silicon branch of the first device, so in all consecutive tests, only the oscillating lower branch was held at the ETT, with the instrument branches open.

Phase two: physical model

**Figure 1:**
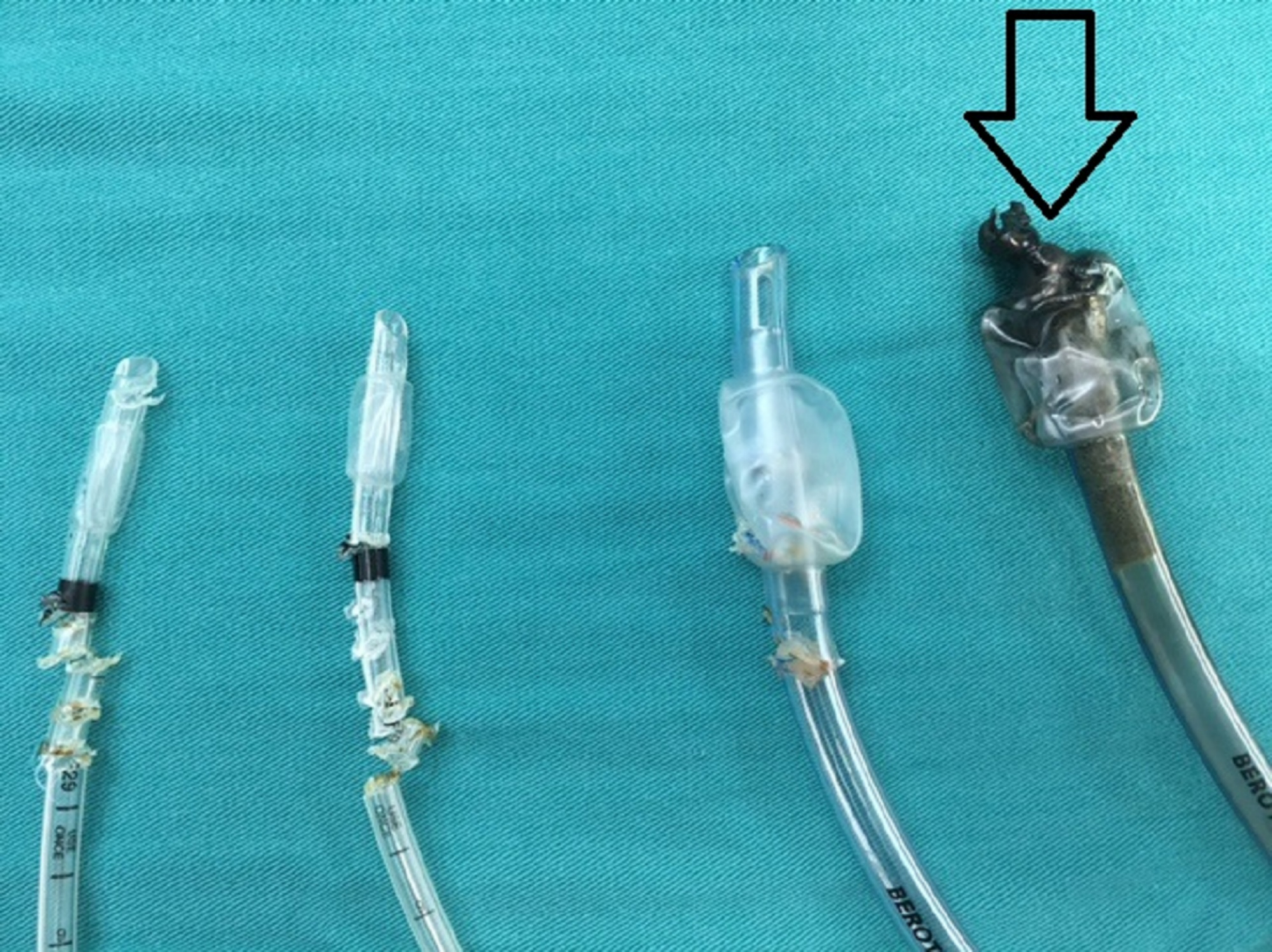
The Ultracision Harmonic forceps cut the intubation tubes and the charred intubation tube from the electrocautery attempt (arrow). Ultracision Harmonic: Ethicon Endosurgery, Cincinnati, Ohio, USA

In this half-closed, oxygen-enriched cavity again, no ignition or sustained fire could be produced neither on the chicken tissue in proximity with the outlet of the ETT nor at any location of the ETT. The first structure attacked - the cuff - was perforated immediately after the activation of the Harmonic Ace Shears on it. Then, the ETT was cut at several instances, each time proximally, without signs of ignition. Again, visible aerosols developed in a quantity that did not impair the intracavitary visibility significantly. The cutting lines at the ETT were partially brownish (Figure 1). An attempt at remote temperature measurement failed.

Phase three: verification of the physical model

**Figure 2:**
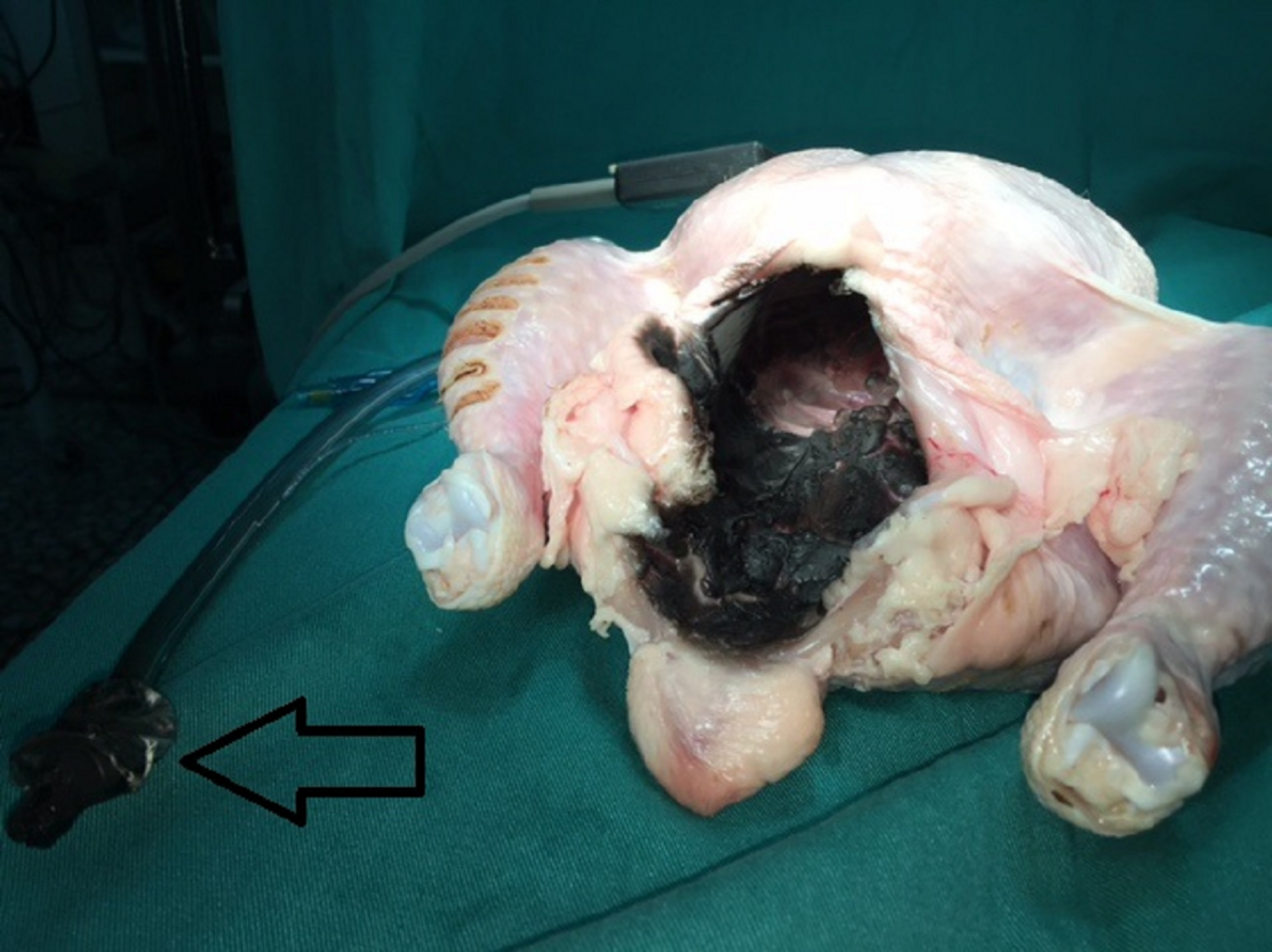
The charred avian model and intubation tube (arrow) after the electrocautery ignition.

In each of the chickens used for the physical model of interaction of the Harmonic Ace Shears with the ETT, the electrosurgical device was used. A fire was ignited when tissue electrocautery was performed a few millimeters from the opening of the ETT (Figure 2). The time to ignition was either nine or 12 seconds. The burning, with a visible flame, involved not only the tissues in contact with the Bovie but almost the whole internal surface of the degutted row chicken. Abundant black smoke developed, which impaired visibility. After ignition, the oxygen supply was immediately discontinued for safety reasons.

## Discussion

Surgery-related ignition of the plastic ETT in the upper airways is a known and potentially devastating event that is extensively reported and analyzed in the literature [3]. Fire is defined as a process involving rapid oxidation at elevated temperatures accompanied by the evolution of the heated gaseous products of combustion and the emission of visible and invisible radiation.

Two modes of combustion are recognized - the flaming and the non-flaming (smoldering or glowing). The concept of fire may be pictured with the Fire Tetrahedron, consisting of oxygen to sustain combustion, sufficient heat to raise the material to its ignition temperature, fuel or combustible material, and subsequently, an exothermic chemical chain reaction in the material. This represents a refined development of the Fire Triangle [13]. The presence of air/oxygen flow (which is the case in mechanical ventilation) may cause smoldering to turn into flaming.

The majority of the cases of OR fires are attributed to endoscopic oral/pharyngeal/laryngeal surgery of tracheostomy. More than half of the cases appear during intracavitary surgery in the pharynx and the airways and less during transcutaneous surgery on the body surface [12].

The main surgical tools related to the risk of ignition include monopolar and occasionally bipolar diathermy, lasers (predominantly C0_2_), and light cords [14]. Any other surgical tools to be used in this area should be evaluated for their specific risk profile to cause accidental fires in contact with the ETT, other plastic tubings (nasogastric tubes, retracting loops), and other surgical materials in the presence of oxygen or other gaseous oxidizing agents.

The harmonic scalpel is an optional surgical cutting tool, which fights its way in otorhinolaryngology and head and neck surgery as an alternative to the most commonly used one - the monopolar cautery [4]. Initially developed for laparoscopic surgery in the 1960s, ultrasonic dissection has been used in a growing number of surgical procedures, including the head and neck region. In the area of the pharynx and larynx, the tool has been evaluated so far for tongue resection [4-5], tonsillectomy [6], sphincter pharyngoplasty [7], oropharyngeal cancer resection [8], uvulopalatopharyngoplasty [9], and endoscopic repair of Zenker's diverticulum [15] and for some open surgeries in close proximity to ETT as open (pharyngo) laryngectomies [16]. As with all high-energy surgical tools, a harmonic scalpel causes a local rise in the temperature.

The mean peak temperatures at the jaws of the ultrasonic instrument are proportional to the activation time and power setting and may reach 297°C under extreme operating conditions [17]. The eventual friction over the hard solid coil of a wire-reinforced ETT may result in higher jaw temperature [18]. The harmonic scalpel is known to elevate the temperature of the nearby tissues by more than 40°C [19]. The thermal parameters of new and reprocessed harmonic scalpels do not appear to differ [20].

Histologic studies show the advantages of low-thermal-injury devices (harmonic scalpel, CO_2_ laser) against monopolar cautery for tissue preservation both at the specimen margin and the oropharyngeal resection site [4,21]. In the same time, both monopolar cautery and CO_2_ laser obviously represent an ignition hazard in proximity with the tracheal tubes. The risk profile of the harmonic scalpel with regards to ignition and fire has still not been thoroughly evaluated. Only one report briefly states that the harmonic scalpel is advantageous in terms of fire safety in the OR for tracheotomies and cricothyroidotomies [10]. However, this has not been tested in a standardized systematic setting.

There are occasional reports of ETT-related incidents in relation to ultrasonic surgical devices. A case of cut inflation cuff tube during adult tonsillectomy with Harmonic Scalpel resulting in a massive air leak was reported [2]. In another case, the main body of the ETT was perforated with the Harmonic Scalpel during oropharyngeal oncologic resection on a nasotracheally intubated patient, again manifested by an air leak [22]. Penetrating injuries of wire-reinforced ETT caused by a harmonic scalpel has also been described during uvulopalatopharyngoplasty. In all these cases, there were no signs of ignition. In their report, Coulson and Bakhshay describe superficial and penetrating injuries to ETT but without information on the presence of oxygen and the exact description of their experimental setting [10]. In all published reports, the ETT damage by ultrasonic scalpels resulted in air leak without ignition.

The potentially combustible materials and devices in an OR that may play the role of fuel have different inflammability and burnability. An important characteristic of every material is its autoignition temperature (kindling point). This is the lowest temperature at which it will spontaneously ignite in a normal atmosphere without an external source of ignition such as a flame or spark. For the rigid PVC (unplasticized, PVC-U) the autoignition temperature is 450°C while the flash ignition temperature is 390°C. The ignition resistance of common flexible PVC formulations is lower, but with specialized formulations, it may be significantly increased. So PVC for medical use appears to be a heterogeneous group, with additives quite depending on the manufacturer of the ETT [18,23]. In this way, it is impossible to state that all ETTs will be equally ignition- and fire-resistant and will behave in the same way in contact with an Ultracision scalpel.

The avian model adopted for our experiment is well-established for simulating an injury of the ETT in a body cavity, resembling clinical conditions in oral and pharyngeal surgery. The results from our phase three test (verification of the physical model) clearly showed that the physical model selected is reliable and easily repeatable, with similar qualitative and quantitative characteristics, as originally published [24].

## Conclusions

Ultracision Harmonic forceps appear to be aggressive to PVC-ETTs, as they easily penetrate their wall, damage the inflation cuff of the tube, create large openings in the tubings, or may even completely transect them. Ignition of the ЕТТ is impossible in the worst-case circumstances simulated. Ultracision Harmonic forceps appear to be a surgical dissecting, resecting, and coagulating tool that eliminates the risk of an ETT fire in pharyngeal and laryngeal surgery, especially in comparison with other long-established tools such as electrocautery and CO_2_ laser.
